# LncRNA-AC068228.1 Is a Novel Prognostic Biomarker That Promotes Malignant Phenotypes in Lung Adenocarcinoma

**DOI:** 10.3389/fonc.2022.856655

**Published:** 2022-02-23

**Authors:** Xiulin Jiang, Min Chen, Junyi Du, Hong Bi, Xiang Guo, Chao Yang, Xu He, Zhixian Jin

**Affiliations:** The Department of Pneumology, The First People’s Hospital-Calmette Hospital of Kunming, Kunming, China

**Keywords:** lncRNA, lung adenocarcinoma, immune cell infiltration, prognosis biomarker, malignant phenotypes

## Abstract

**Background:**

The crucial roles played by lncRNA-AC068228.1 in primary malignant cancer remain poorly understood. This study aimed at examining the clinical significance and evaluating the biological function of AC068228.1 in lung adenocarcinoma (LUAD).

**Methods:**

We used data obtained from The Cancer Genome Atlas (TCGA), Genotype-Tissue Expression (GTEx), and the Gene Expression Omnibus (GEO) database to examine the expression of AC068228.1 in LUAD patients, and the prognostic and diagnostic value of those levels. Functional experiments were conducted to determine the function of AC068228.1 on LUAD cells. Signaling pathway enrichment analysis of AC068228.1 was conducted using the clusterProfiler and Gene Set Enrichment Analysis (GSEA) software. We analyzed the correlation between AC068228.1 expression and immune infiltration level in LUAD using the single-sample gene set enrichment analysis (ssGSEA) method by the R package GSVA.

**Results:**

AC068228.1 expression was significantly elevated in LUAD tissues compared with normal tissues. Higher expression of AC068228.1 was strongly correlated with adverse clinical outcomes and was identified as an independent prognostic marker for LUAD patients. GSEA and infiltration analysis confirmed that AC068228.1 expression was significantly correlated with immune cells infiltrating in LUAD. Knockdown of AC068228.1 inhibited the cell proliferation and cell migration of LUAD.

**Conclusions:**

AC068228.1 was upregulated in LUAD and was significantly correlated with adverse clinical outcomes. Meanwhile, it was associated with immune cell infiltration and could be used as a promising diagnostic and prognostic biomarker for LUAD patients.

## Introduction

Lung cancer is the leading cause of cancer-related morbidity and mortality worldwide (1). As the tumor with the highest mortality, lung cancer mainly includes small cell lung cancer and non-small cell lung cancer (NSCLC), the NSCLC is composed of adenocarcinoma (LUAD), squamous cell carcinoma (LUSC), and large-cell carcinoma (LCC) (2). With the progress of science and technology and the diversification of medical means, the prognosis of lung cancer patients has been improved to a certain extent, while most lung cancer patients have a poor overall 5-year survival rate of ~5% (3). Thus, elucidating the complex molecular mechanism underlying lung cancer progression and identifying the key molecules regulating cancer progression is crucial for the treatment of lung cancer.

LncRNAs are a kind of ncRNAs whose transcripts with a length of more than 200 nucleotides do not have a protein coding potential. Mounting evidence has demonstrated that lncRNA abnormal expression and overactivation are usually involved in the cancer initiation and progression (4). LncRNAs can modulate gene expression *via* influencing the structure of chromatin (5, 6), histone modification (7), and sponging of microRNA (8). Aberrantly expressed lncRNAs have been reported to correlate with the development and progression of lung cancer (9). For example, Gong et al. found that lncRNA JPX was highly expressed in lung cancer and correlated with the tumor size and an advanced stage. Forced expression of JPX facilitated lung cancer cell proliferation *in vitro* and facilitated lung tumor growth *in vivo* (10). Lu et al. reported that lnc-IGFBP4-1 was overexpressed in lung cancer tissues and its higher expression is associated with TNM stage and lymph node metastasis. Depletion of lnc-IGFBP4–1 significantly inhibited cell proliferation and induced apoptosis. Further research showed that lnc-IGFBP4-1 *via* affecting the expression of HK2, PDK1, and LDHA led to enhancement of the ATP production level and involvement in lung cancer progression (11). Furthermore, lncRNA AFAP1-AS1 was found to modulate NSCLC cell proliferation *via* interacting with EZH2 and recruiting EZH2 to the promoter regions of p21, thus inhibiting p21 expression (12).

In our previous study, we developed a new method called CVAA (Cross-Value Association Analysis), which functions without a normalization and distribution assumption. We applied it to large-scale pan-cancer transcriptome data generated by The Cancer Genome Atlas (TCGA) project and successfully discovered numerous new differentially expressed genes (DEGs), including AC068228.1 (ENSG00000253258), which are mainly located in chromosomes chr8:123153966–123273028 and are 777 nucleotides in length. However, the role played by AC068228.1 in LUAD remains poorly understood. In the present study, we examine the expression level of AC068228.1 in LUAD tissues and LUAD cell lines. Moreover, LUAD expression and clinical data from TCGA were downloaded and utilized to assess the prognostic and diagnostic value of AC068228.1 and the correlation between AC068228.1 expression and diverse clinical features in LUAD patients. GSEA was conducted to determine the AC068228.1-related signaling pathways involved in LUAD. The correlation between AC068228.1 expression and immune cell infiltrate in LUAD was determined using the ssGSEA method. Finally, we determine the biological effects of AC068228.1 on LUAD cell lines by employing various biological function experiments.

## Materials and Methods

### Data Collection

TCGA-LUAD cohort data and corresponding clinical information of 535 LUAD patients were downloaded from the TCGA website (https://portal.gdc.cancer.gov/repository). LUAD patients were classified into low- and high-AC068228.1 expression groups according to the median AC068228.1 expression value. AC068228.1 expression data from datasets GSE81089 were downloaded from the GEO database and validated for expression analyses. The gene expression profiles were normalized using the scale method provided in the “limma” R package. Data analysis was performed with the R (version 3.6.3) and ggplot2 [3.3.3] packages. The expression data were normalized to transcripts per kilobase million (TPM) values before further analysis. Besides, the receiver operating characteristic (ROC) curve was used to evaluate the diagnostic value of AC068228.1 using the pROC R package and ggplot2 R package.

### Nomogram Construction and Evaluation

Based on the multivariate Cox analysis results, we established a nomogram to predict the prognosis of lung adenocarcinoma patients. According to the prognosis model, we calculated each patient’s risk score as the total score of each parameter, which could predict the prognosis of lung adenocarcinoma patients (13).

### Gene Set Enrichment Analysis

We utilized the GSEA software to analyze the potential signaling pathway and molecular function in lung adenocarcinoma (14, 15). A customized Perl script was used to perform GSEA between high-AC068228.1 and low-AC068228.1 groups. According to the default statistical methods, an adjusted p-value < 0.05 was considered significant.

### Immune Infiltration Analysis by ssGSEA

We used a GSVA R package to examine the lung adenocarcinoma immune infiltration of 24 tumor-infiltrating immune cells in tumor samples through ssGSEA (16, 17). The correlation between AC068228.1 and infiltration levels of immune cells was analyzed by the Spearman’s correlation, and these immune cells with the different expression groups of AC068228.1 were analyzed by the rank-sum test.

### Patients and Samples

All primary LUAD samples were obtained from patients at the First People’s Hospital-Calmette Hospital of Kunming, China. All samples were instantly submerged in RNA later upon collection. All samples were collected from patients who provided informed consent under institutional review board-approved protocols and were stored at −80°C until use.

### Cell Culture

The BEAS-2B cell line was purchased from the Cell Bank of Kunming Institute of Zoology and cultured in BEGM media (Lonza, CC-3170). Lung cancer cell lines, including A549, HCC827, H1299, and H1975, were purchased from Cobioer, China, with STR documents, and were cultured in RPMI-1640 medium (Corning) supplemented with 10% fetal bovine serum (FBS) and 1% penicillin/streptomycin.

### Constructs, Lentiviral Preparation, and Establishment of Different Cell Lines

For shRNA knockdown experiments, independent shRNAs targeting to different regions of AC068228.1 RNA were constructed using a pLKO.1 vector (Addgene, Watertown, MA, USA), and the oligo sequences were provided in the following. Lentiviruses were generated according to the manufacturer’s protocol as previously documented (10) and indicated that cells were infected by viruses twice with 48- and 72-h viral supernatants containing 4 μg/ml polybrene, and stable cell lines were established by appropriate puromycin selection. The two independent AC068228.1 targeting sequences are shRNA, 5′-GGGTGATGGTGCCAAATATAT-3′.

### Cell Proliferation Assays

For growth curve assay, 8 × 10^3^ cells were plated onto 12-well plates, and the cell numbers were subsequently counted each day using an automatic cell analyzer Countstar (Shanghai Ruiyu Biotech Co., Shanghai, China). For colony formation assay, a total of 500 cells/well was plated onto 6-well plates and cultured for 2 weeks at 37°C. The medium was changed every 3 days. Two weeks later, the indicated cells were fixed with 4% PFA for 30 min at room temperature and subsequently stained with 0.1% crystal violet for 30 min at room temperature.

### Cell Migration Assays

Cell migration assays were performed as previously documented (11). Cell migration assay was performed as previously described (10). Briefly, indicated cells were seeded into 6-well plates (9 × 10^5^/cell) and incubated for 1 day, and then a straight line was scraped with pipette tips. Detached cells were removed. Photographs were taken at the indicated time, and the relative traveled distance was measured. For the transwell migration assay, 2.5 × 10^4^ cells/well in 100 μl serum-free medium were plated in a 24-well plate chamber insert, and the lower chamber was filled with 10% FBS. After incubation for 24 h, cells were fixed with 4% PFA, washed, and then stained with 0.5% crystal violet for further capturing of pictures.

### Real-Time RT-PCR Assay

The real-time RT-PCR assay, cells were lysed by RNAiso Plus (Takara Bio, Beijing, China, Cat. 108-95-2). Total RNAs were extracted according to the manufacturer’s protocol and then reverse transcribed by using the RT Reagent Kit (Takara Bio, Beijing). The primers used in this study are as follows: β-actin-F: AAGTGTGACGTGGACATCCGC, β-actin-R: CCGGACTCGTCATACTCCTGCT, AC068228.1-F: TACCGCTGTCCTGAGCAATG, AC068228.1-R: CCTTCCCGTTTCTCTTCCCC. β-Actin RNA was used as internal control, and the 2-ΔΔ Ct method was utilized to calculate the relative expression of AC068228.1.

### Statistical Analysis

For the datasets from the TCGA database, statistical analyses were performed using R. The Wilcoxon rank-sum test and chi-square test were used to estimate the association between AC068228.1 and clinical pathologic characteristics. The Kaplan–Meier method was used to calculate LUAD patient survival rates. Univariate and multivariate Cox analyses were performed to assess the correlation between clinical features and overall survival, disease-free survival, and progression-free survival. For the data regarding the function of AC068228.1, GraphPad Prism 7.0 was used for statistical analyses. The Student’s t-test evaluated the statistical significance between groups. The significance of the data between two experimental groups was determined by Student’s *t*-test, and multiple-group comparisons were analyzed by one-way ANOVA. *p* < 0.05 (*), *p* < 0.01 (**), and *p* < 0.001 (***) were significant.

## Results

### AC068228.1 Was Overexpressed in Lung Adenocarcinoma

**Figure 1:**
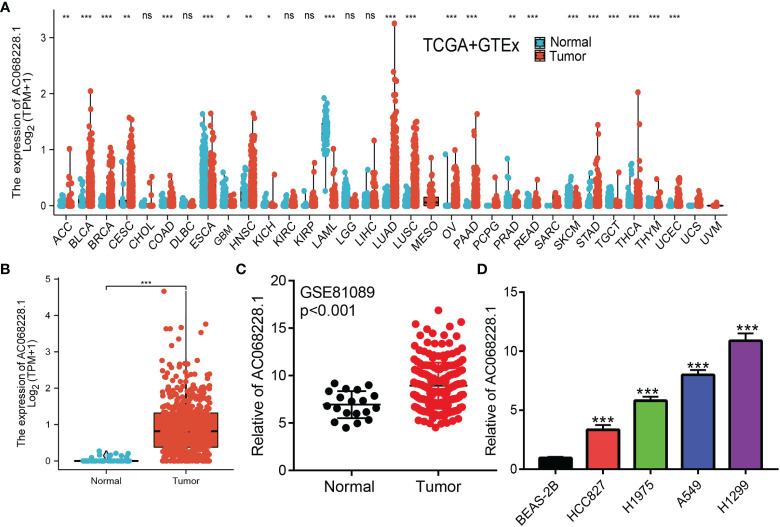
Expression level and prognosis of AC068228.1 in human cancer. **(A)** Expression of AC068228.1 in normal and tumor tissues in TCGA and GTEx datas. **(B)** Expression of AC068228.1 in LUAD based on the TCGA dataset. **(C)** Expression levels of AC068228.1 in lung cancer based on the GEO dataset. **(D)** The relative expression level of AC068228.1 in lung adenocarcinoma cancerous cell lines, including A549, H1975, HCC827, and H1299 examined by real-time RT-PCR, compared to normal human bronchial epithelial cell line: BEAS-2B. *p < 0.05, **p < 0.01, and ***p < 0.001.

We conducted pan-cancer analyses utilized the Wilcoxon rank-sum test to compare AC068228.1 expression in normal tissues and tumor samples using RNA sequencing data obtained from TCGA and GTEx databases. Results showed that AC068228.1 was highly expressed in 15 tumor types, including adrenocortical carcinoma (ACC), bladder urothelial carcinoma (BLCA), breast invasive carcinoma (BRCA), cervical squamous cell carcinoma and endocervical adenocarcinoma (CESC), colon adenocarcinoma (COAD), head and neck squamous cell carcinoma (HNSC), lung adenocarcinoma (LUAD), lung squamous cell carcinoma (LUSC), ovarian serous cystadenocarcinoma (OV), pancreatic adenocarcinoma (PAAD), rectum adenocarcinoma (READ), stomach adenocarcinoma (STAD), thyroid carcinoma (THCA), thymoma (THYM), and uterine corpus endometrial carcinoma (UCEC) (**Figure 1A**). Furthermore, significantly higher levels of AC068228.1 expression were found in the LUAD tissues when compared with the normal tissues (**Figure 1B**). AC068228.1 was upregulated in LUAD in GSE81089 datasets, which is consistent with the TCGA database we discovered (**Figure 1C**). Finally, we found that AC068228.1 was overexpressed in LUAD cell lines than in the normal lung epithelial cell line (**Figure 1D**).

### Overexpression of AC068228.1 Was Associated With Adverse Clinical Parameters in Lung Adenocarcinoma

**Figure 2:**
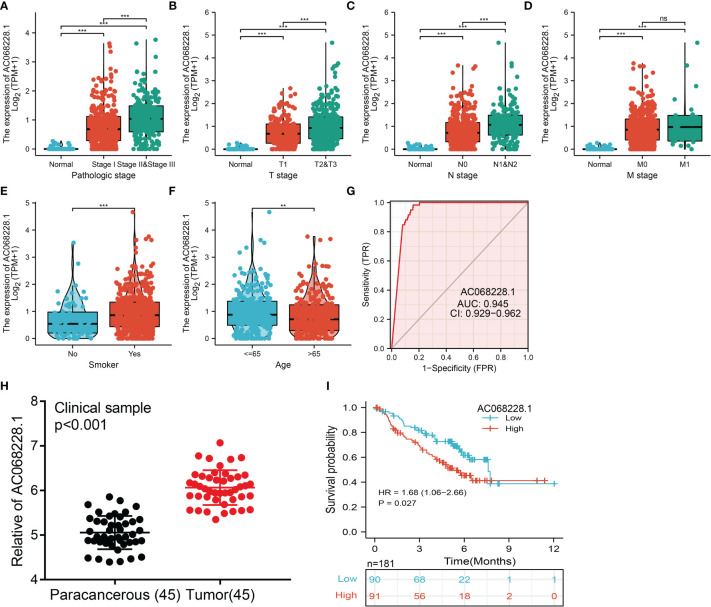
Clinical significance of lncRNA-AC068228.1 in lung adenocarcinoma. **(A–F)** Correlation between AC068228.1 expression and clinical parameters include pathological, TNM stages, smoker, and age. **(G)** ROC curves were used to determine the diagnostic value of AC068228.1 in lung adenocarcinoma based on TCGA-LUAD. **(H, I)** The expression level and prognostic value of AC068228.1 in LUAD validation by our clinical samples NS: P > 0.05, **p < 0.01, ***p < 0.001.

To determine the correlation between AC068228.1 expression and diverse clinical parameters in lung adenocarcinoma, we downloaded the expression data and the clinical characteristics of 513 LUAD patients from the TCGA cohort. The LUAD patients were divided into two groups (a high and low expression group AC068228.1, respectively) according to the median value for AC068228.1 expression. We found that the parameters of pathologic stage, TNM stage, age, and smoker were significantly correlated with AC068228.1 expression (**Figures 2A–F**). We undertook analysis of receiver operating characteristic (ROC) curves of AC068228.1 expression to obtain the area under the ROC curve (AUC) values (0.945) (**Figure 2G**). To further validate the correlation between AC068228.1 expression and overall survival, we examined the expression and prognostic value of AC068228.1 in LUAD by clinical samples from our Hospital (N = 181). The results also showed that AC068228.1 was highly expressed in LUAD and correlated with adverse clinical outcomes (**Figures 2H, I**). These results showed that AC068228.1 could be used as a biomarker to diagnose LUAD with high sensitivity and specificity.

### Prognostic Value of AC068228.1 in LUAD

**Figure 3:**
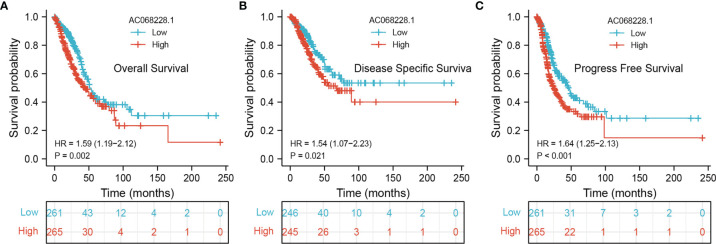
Kaplan–Meier survival analyses for prognostic values of AC068228.1 in LUAD. **(A–C)** Kaplan–Meier survival curves showed that lung adenocarcinoma patients with high AC068228.1 expression exhibited poor overall survival, disease-specific survival, and progression-free survival based on the TCGA-LUAD dataset.

To ascertain the prognostic role of AC068228.1 in LUAD, Kaplan–Meier analysis evaluated the prognostic value of AC068228.1 in the AC068228.1-high and AC068228.1-low groups. The results confirmed that LUAD patients with higher levels of AC068228.1 expression correlated with poor overall survival (OS), disease-specific survival (DSS), and progression-free survival (PFS) (**Figures 3A–C**).

Next, univariate and multivariate Cox regression analyses were conducted to investigate factors that correlated with a patient’s overall survival (OS), disease-specific survival (DSS), and progression-free survival (PFS) (**Table 1**). The univariate analysis confirmed that pathologic stage, TNM stage, and AC068228.1 expression were significantly associated with the overall survival time of LUAD patients. A multivariate analysis showed that AC068228.1 expression and T stage independently predicted overall survival time (**Table 1**). Furthermore, the univariate analysis confirmed that pathologic stage, TNM stage, and AC068228.1 expression were significantly associated with the disease-specific survival time of LUAD patients. A multivariate analysis showed that AC068228.1 expression independently predicted disease-specific survival time (**Table 2**). Finally, the univariate analysis confirmed that pathologic stage, T stage, and AC068228.1 expression were significantly associated with the progression-free survival time of LUAD patients. A multivariate analysis showed that AC068228.1 expression and T stage independently predicted progression-free survival time (**Table 3**). These results confirmed that AC068228.1 could serve as an independent prognostic factor for LUAD patients.

**Table 1:** Univariate and multivariate Cox regression analyses of different parameters on overall survival in lung adenocarcinoma.

Characteristics	Total (N)	Univariate analysis		Multivariate analysis	
		Hazard ratio (95% CI)	p value	Hazard ratio (95% CI)	p value
T stage	523				
T1 and T2	457				
T3 and T4	66	2.317 (1.591-3.375)	<0.001	1.713 (1.066-2.753)	0.026
N stage	510				
N0 and N1	437				
N3 and N2	73	2.321 (1.631-3.303)	<0.001	1.273 (0.608-2.664)	0.522
Pathologic stage	518				
Stage II and stage I	411				
Stage IV and stage III	107	2.664 (1.960–3.621)	<0 .001	1.774 (0.815–3.862)	0.149
M stage	377				
M0	352				
M1	25	2.136 (1.248–3.653)	0.006	1.173 (0.523–2.632)	0.699
AC068228.1	526	1.619 (1.339–1.957)	<0.001	1.414 (1.126–1.775)	0.003

**Table 2:** Univariate and multivariate Cox regression analyses of different parameters on disease-specific survival in lung adenocarcinoma.

Characteristics	Total (N)	Univariate analysis		Multivariate analysis	
		Hazard ratio (95% CI)	p value	Hazard ratio (95% CI)	p value
T stage	488				
T1 and T2	430				
T3 and T4	58	1.974 (1.190–3.275)	0.008	1.516 (0.772–2.974)	0.227
N stage	475				
N0 and N1	410				
N3 and N2	65	1.971 (1.247–3.115)	0.004	1.295 (0.433–3.879)	0.644
Pathologic stage	483				
Stage II and stage I	389				
Stage IV and stage III	94	2.436 (1.645–3.605)	< 0.001	1.489 (0.466–4.752)	0.502
M stage	344				
M0	323				
M1	21	2.455 (1.269–4.749)	0.008	1.785 (0.540–5.905)	0.343
AC068228.1	491	1.663 (1.311–2.110)	< 0.001	1.493 (1.116–1.997)	0.007

**Table 3:** Univariate and multivariate Cox regression analyses of different parameters on progression-free survival in lung adenocarcinoma.

Characteristics	Total (N)	Univariate analysis		Multivariate analysis	
		Hazard ratio (95% CI)	p value	Hazard ratio (95% CI)	p value
T stage	523				
T1 and T2	457				
T3 and T4	66	1.811 (1.249-2.628)	0.002	1.656 (1.102-2.487)	0.015
N stage	510				
N0 and N1	437				
N3 and N2	73	1.325 (0.914–1.919)	0.137		
Pathologic stage	518				
Stage II and stage I	411				
Stage IV and stage III	107	1.513 (1.105–2.071)	0.010	1.164 (0.817–1.656)	0.400
M stage	377				
M0	352				
M1	25	1.513 (0.855–2.676)	0.155		
AC068228.1	526	1.509 (1.262–1.804)	< 0.001	1.495 (1.242–1.800)	< 0.001

### Prognostic Role of AC068228.1 in Subgroup Analyses

**Figure 4:**
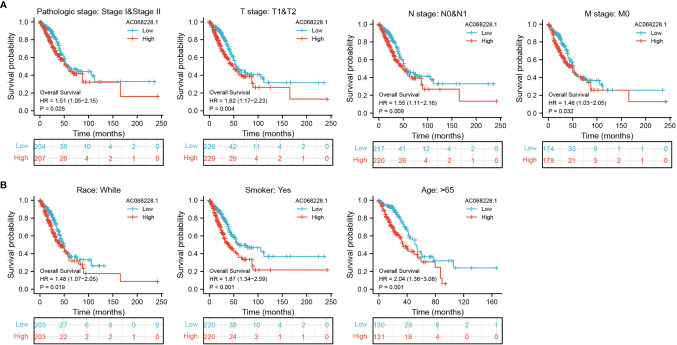
Kaplan–Meier survival analyses for prognostic values of AC068228.1 in different subgroups stratified by clinical features. **(A, B)** Kaplan–Meier curves for overall survival of AC068228.1 in subgroups including stage I-II, T1-T2, N0-N1, M0, Race, white, age>65years, and Smoker in TCGA-LUAD cohort.

To better determine the factors that affect the prognosis of LUAD patients, we performed stratification analyses by creating survival charts. These results confirmed that increased AC068228.1 expression was strongly associated with shorter overall survival among patients with pathologic stage, tumor node metastasis (TNM) stage, race, smoker, and age (**Figures 4A, B**).

### Construction and Validation of AC068228.1-Based Nomogram

**Figure 5:**
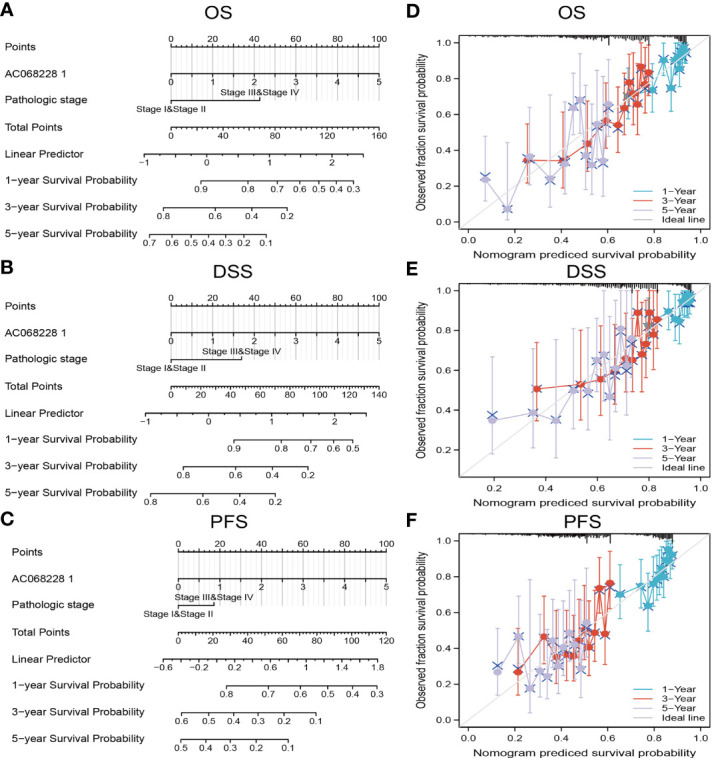
Construction and performance validation of the AC068228.1-based nomogram for lung adenocarcinoma patients. Nomogram used to predict overall survival, disease-free survival, and progression-free survival of lung cancer patient **(A–C)**. The calibration curve and Hosmer–Lemeshow test of nomograms in the TCGA-lung adenocarcinoma cohort for **(D)** overall survival, **(E)** disease-specific survival, and **(F)** progression-free survival.

The multivariate analysis result confirmed that AC068228.1 is an independent prognostic factor in LUAD. We then constructed a prediction model for overall survival, disease-free survival, and progression-free survival by integration AC068228.1 expression and pathologic stage. We established a nomogram to integrate AC068228.1 as a LUAD biomarker; higher total points on the nomogram for overall survival, progression-free survival (PFS), and disease-specific survival respectively indicated a worse prognosis (**Figures 5A–F**). In summary, these results indicated that the nomogram could provide a relatively accurate prediction of LUAD patients’ survival time.

### KEGG and GO Enrichment Analysis

**Figure 6:**
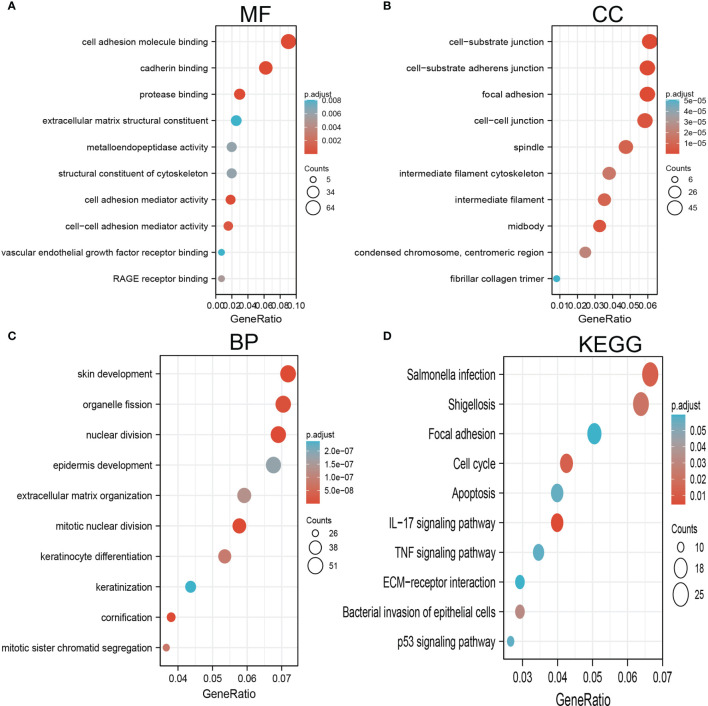
GO and KEGG enrichment analysis for AC068228.1. **(A)** Top 10 enrichment terms in MF categories in LUAD. **(B)** Top 10 enrichment terms in CC categories in LUAD. **(C)** Top 10 enrichment terms in BP categories in LUAD. **(D)** Top 10 KEGG enrichment pathways in LUAD.

By using Spearman correlation analysis, we obtained 200 genes that positively correlated with AC068228.1 expression in the TCGA-LUAD cohort (**Supplementary Table 1**). We utilized the R package clusterProfiler-conducted enrichment analysis of AC068228.1 positively related genes involved in several biological processes (BPs), molecular functions (MFs), cellular components (CCs), and Kyoto Encyclopedia of Genes and Genomes (KEGG). The results confirmed that the genes for MFs were mainly involved in the cell adhesion molecule binding, cadherin binding, protease binding, extracellular matrix structural constituent, metalloendopeptidase activity, structural constituent of cytoskeleton, cell adhesion mediator activity, cell–cell adhesion mediator activity, vascular endothelial growth factor receptor binding, and RAGE receptor binding (**Figure 6A**). The genes for CCs and BP were mainly involved with cell–substrate junction, cell–substrate adherens junction, intermediate filament cytoskeleton, skin development, epidermis development, extracellular matrix organization, mitotic nuclear division, and keratinocyte differentiation (**Figures 6B, C**). Moreover, KEGG enrichment analysis revealed that these genes were predominately correlated with the focal adhesion, cell cycle, apoptosis, IL-17 signaling pathway, TNF signaling pathway, extracellular matrix (ECM)–receptor interaction, and p53 signaling pathway (**Figures 6D**).

### AC068228.1-Related Signaling Pathways Based on Gene Set Enrichment Analysis

**Figure 7:**
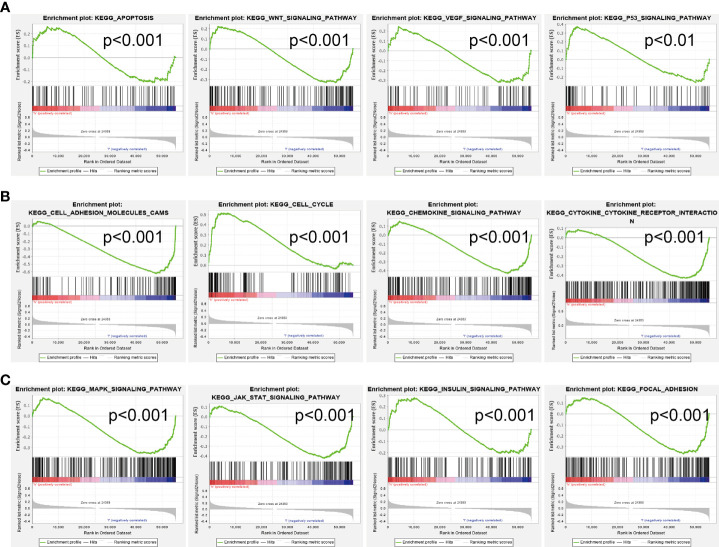
Identification of AC068228.1-related signaling pathways in lung adenocarcinoma. **(A–C)** Top 12 significant KEGG pathways associated with AC068228.1 examined by GSEA software.

We conducted GSEA to explore the potential signaling pathway in the high AC068228.1 expression groups of LUAD patients. We selected the top 12 datasets with a high normalized enrichment score (NES) (**Figures 7A–C**) . The results revealed that apoptosis, Wnt signaling pathway, VEGF signaling pathway, P53 signaling pathway, cell adhesion molecules (cams), cell cycle, chemokine signaling pathway, cytokine–cytokine receptor interaction, MAPK signaling pathway, JAK-STAT signaling pathway, insulin signaling pathway, and focal signaling pathway were significantly enriched in the KEGG pathway (**Figures 7A–C**).

### Correlation Between AC068228.1 Expression and Immune Infiltration

**Figure 8:**
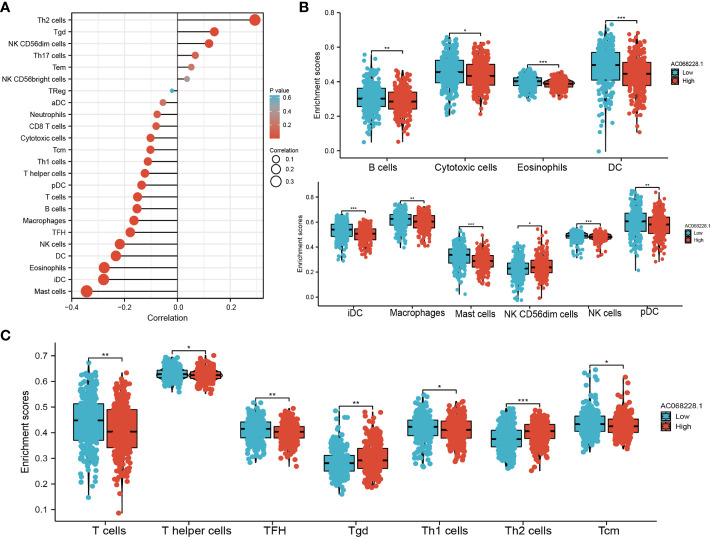
Correlation analysis of AC068228.1 expression and infiltration levels of immune cells in LUAD tissues. **(A)** Correlation between the relative abundances of 24 immune cells and lncRNA AC068228.1 expression level. **(B, C)** Box plots of the correlations between AC068228.1 or molecular model expression and infiltration levels of immune cells. *p < 0.05, **p < 0.01, ***p < 0.001.

SsGSEA with Spearman’s rank correlation was utilized to determine the correlation between AC068228.1 expression and infiltration levels of 24 immune cell types (**Figure 8A**). The results suggested that AC068228.1 expression was positively correlated with Th2 cell, Tgd, and NK CD56dim cells infiltration but negatively associated with Th1 cells, T helper cells, pDC, T cells, B cells, macrophages, follicular helper T (TFH), NK cells, DC, eosinophils, iDC, and mast cells in LUAD (**Figure 8A**). Our study indicated that the expression of AC068228.1 was positively or negatively associated with these immune cell types (**Figures 8B, C**).

### AC068228.1 Promotes Proliferation, Migration, and Invasion of LUAD Cells

**Figure 9:**
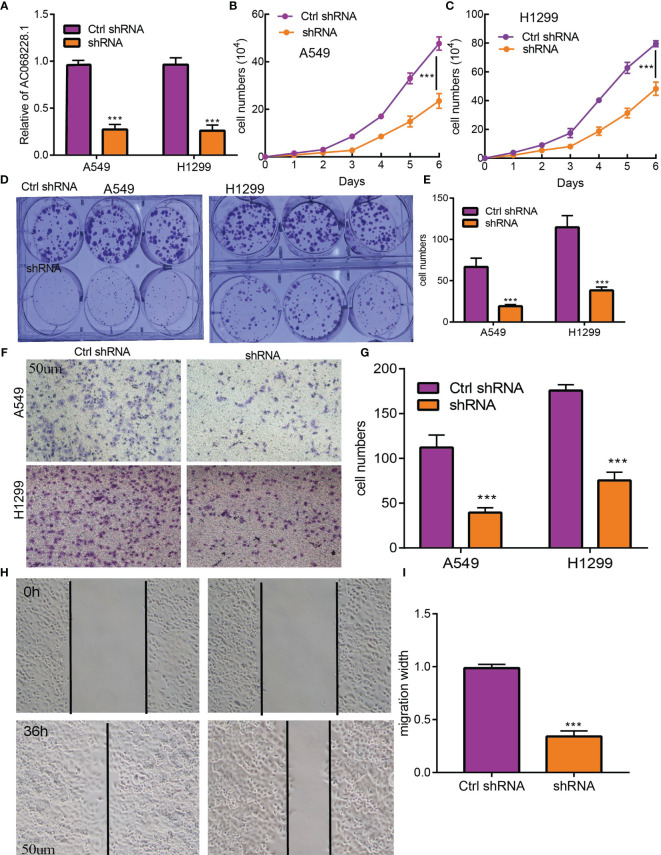
AC068228.1 promotes LUAD cell proliferation, migration, and invasion *in vitro*. **(A, B)** Establishment of AC068228.1 knockdown cell lines in A549 and H1299 cells verified by real-time RT-PCR. **(C–E)** Knockdown of AC068228.1 significantly inhibits cell proliferation in A549 and H12995 cells as measured by a growth curve and colony formation. **(F–I)** knockdown of AC068228.1 dramatically inhibits A549 and H1299 cell migration ability examined by transwell and wound healing assays. ***p < 0.001.

To examine the biological function of AC068228.1 on LUAD cells, we reduced the AC068228.1 levels in LUAD cells by transfection with shRNA for AC068228.1. Knockdown efficiency was determined by RT-qPCR assay (**Figure 9A**). Cell proliferation and colony formation assays were conducted to determine the effect of AC068228.1 on LUAD cell proliferation. The results confirmed that reduced AC068228.1 levels significantly inhibited the proliferation of LUAD cells (**Figures 9B–E**). Furthermore, Transwell and wound healing assays were conducted to explore how AC068228.1 affected the metastatic ability of LUAD cells. Knockdown of AC068228.1 levels in the A549 and H292 cell lines notably inhibited the migration of LUAD cells (**Figures 9F–I**). These results showed that AC068228.1 could promote the cell proliferation and migration of LUAD.

## Discussion

Lung cancer is the highest mortality and incidence rate in all cancers, resulting in a huge economic burden, and remains a huge threat to public health (2). With the progress of science and technology and the diversification of medical means, the prognosis of lung cancer patients has been improved to a certain extent, while most lung cancer patients have a poor overall 5-year survival rate of ~5% (12). Emerging evidence has demonstrated that lncRNAs exert a crucial effect on multiple physiological processes including cell growth, cell migration, immune response, and lung cancer initiation and progression (18), confirming that lncRNAs could serve as novel biomarkers for diagnosis and predicting the prognosis of lung cancer patients.

In this study, we identify a novel long non-coding RNA (lncRNA, ENSG00000253258), termed AC068228.1. The gene for AC068228.1 is located on chromosomes chr8:123153966–123273028 and are 777 nucleotides in length (**Supplementary Table 2**). No published study has specifically reported the levels of AC068228.1 expression in human cancer, and this is the first study to examine the expression patterns and biological function of AC068228.1 in LUAD. We initially obtained expression data correlated with LUAD from TCGA and conducted a comprehensive bioinformatics analysis for the clinical features and prognosis values of AC068228.1 in LUAD. We found that AC068228.1 was highly expressed in various human cancers, including LUAD cancer tissues, compared to that in adjacent normal tissues. We further used GEO LUAD dataset validation of the above analysis results, which is consistent with the TCGA database we discovered. In addition, we also found elevated levels of AC068228.1 expression in LUAD cell lines when compared with those levels in the BEAS-2B cell line. Furthermore, we found that the parameters of pathologic stage, TNM stage, age, and smoker were significantly correlated with AC068228.1 expression. In our study, we also found that a high expression of AC068228.1 correlated with poor OS, DSS, and PFS in LUAD patients. Multivariate analysis showed that AC068228.1 expression independently predicted OS, DSS, and PFs in LUAD patients. We established a nomogram to integrate AC068228.1 as a LUAD biomarker; higher total points on the nomogram for overall survival, progression-free survival (PFS), and disease-specific survival respectively indicated a worse prognosis. To this end, we performed a comprehensive Kaplan–Meier analysis of a set of subgroups that showed a lower overall survival in the AC068228.1-high groups *versus* the AC068228.1-low groups, including pathologic stage, TNM stage, race, smoker, and age.

It is well established that abnormal epigenetic modification promotes tumor initiation and progression by activating a series of oncogenic signaling pathways (6, 19). To further investigate the function of AC068228.1 in detail, we performed functional annotation based on the enrichment analysis. GO and GSEA analyses revealed that AC068228.1-related genes associated with focal adhesion, cell cycle, apoptosis, IL-17 signaling pathway, TNF signaling pathway, ECM–receptor interaction, and 53 signaling pathway were related to functions that could facilitate carcinogenesis. Finally, our *in vitro* experiments confirmed that AC068228.1 promotes the proliferative and migration abilities of LUAD cells, and all the enrichment results indicated that AC068228.1 was strongly associated with cancer initiation and progression.

The tumor microenvironment plays a central role in many aspects of cellular processes, including cancer progression (20, 21). According to our research, there was a significant negative association between AC068228.1 expression and Th1 cells, T helper cells, pDC, T cells, B cells, macrophages, TFH, NK cells, DC, eosinophils, iDC, and mast cells in LUAD. Therefore, performing a comprehensive analysis of the correlation between AC068228.1 and immune infiltration is warranted.

Although our study has uncovered some new facts, there remain some limitations that should be mentioned. First, our data are only based on public database analysis and lack large clinical samples to validate these analysis results, and further, large-scale samples needed to verify the results would be convincing. Second, the potential molecular mechanism by which AC068228.1 contributes to LUAD carcinogenesis has not been adequately investigated. Finally, we did not conduct the *in vivo* experiments to validate the function of AC068228.1 in the tumor metastasis and tumor microenvironment regulation of LUAD. In the future, we will pay more attention to the function of AC068228.1 in cancer metastasis and tumor microenvironment regulation of LUAD.

## Conclusion

Collectively, our study provides the first evidence that demonstrates the important role played by AC068228.1 in LUAD. We confirmed that an increased level of AC068228.1 expression was significantly correlated with adverse clinical outcomes in LUAD patients. Our multivariate analysis showed that AC068228.1 could serve as an independent diagnostic and prognostic factor. The present study partially illustrates the role played by AC068228.1 in LUAD and suggests its use as a novel diagnostic and prognostic biomarker for LUAD patients.

## Data Availability Statement

The original contributions presented in the study are included in the article/**Supplementary Material**. Further inquiries can be directed to the corresponding author.

## Author Contributions

XLJ and MC designed this work and performed the related assay. JD, HB, XG, CY, and XH analyzed the data. ZXJ supervised and wrote the manuscript. All authors contributed to the article and approved the submitted version.

## Funding

This work was supported by the National Natural Science Foundation of China (82160593, 81560010) and the Science and Technology Planning Project of Yunnan Province [202101AY070001-213, 2017FE467].

## Conflict of Interest

The authors declare that the research was conducted in the absence of any commercial or financial relationships that could be construed as a potential conflict of interest.

## Publisher’s Note

All claims expressed in this article are solely those of the authors and do not necessarily represent those of their affiliated organizations, or those of the publisher, the editors and the reviewers. Any product that may be evaluated in this article, or claim that may be made by its manufacturer, is not guaranteed or endorsed by the publisher.
