# Supplementary material for: LncRNA-AC068228.1 Is a Novel Prognostic Biomarker That Promotes Malignant Phenotypes in Lung Adenocarcinoma
Source: Front Oncol. 2022 Feb 23;12:856655. doi: 10.3389/fonc.2022.856655 (PMC8904746; doi:10.3389/fonc.2022.856655)
Supplement: Supplementary file 1 [file Table_1.docx]

Supplementary Material

**Supplementary Table 1. The top 200 genes that positively correlated with** AC068228.1 in LUAD

| Query | Statistic | P-value | FDR (BH) | Event_SD |
| --- | --- | --- | --- | --- |
| AC068228.1 | 1 | ######## | ######## | 515 |
| LOC100131726 | 0.904376 | ######## | ######## | 515 |
| SLC16A3 | 0.530497 | 1.01E-38 | 6.73E-35 | 515 |
| RHOV | 0.526771 | 4.14E-38 | 2.07E-34 | 515 |
| SEMA4B | 0.513579 | 5.29E-36 | 2.12E-32 | 515 |
| KRT80 | 0.506947 | 5.61E-35 | 1.87E-31 | 515 |
| B4GALT1 | 0.483334 | 1.66E-31 | 4.75E-28 | 515 |
| AHNAK2 | 0.477048 | 1.26E-30 | 3.15E-27 | 515 |
| MYEOV | 0.473268 | 4.18E-30 | 9.27E-27 | 515 |
| CYP24A1 | 0.466989 | 2.96E-29 | 5.91E-26 | 515 |
| GPR115 | 0.465915 | 4.11E-29 | 7.48E-26 | 515 |
| PTGES | 0.463459 | 8.73E-29 | 1.45E-25 | 515 |
| BAG3 | 0.458217 | 4.26E-28 | 6.16E-25 | 515 |
| KRT7 | 0.458172 | 4.31E-28 | 6.16E-25 | 515 |
| BZW1 | 0.450924 | 3.69E-27 | 4.61E-24 | 515 |
| PLIN3 | 0.444617 | 2.29E-26 | 2.54E-23 | 515 |
| TUBB3 | 0.438542 | 1.28E-25 | 1.28E-22 | 515 |
| C1QTNF6 | 0.429925 | 1.39E-24 | 1.27E-21 | 515 |
| CLCF1 | 0.429715 | 1.48E-24 | 1.28E-21 | 515 |
| LRRC59 | 0.429351 | 1.63E-24 | 1.36E-21 | 515 |
| FHL2 | 0.429156 | 1.72E-24 | 1.37E-21 | 515 |
| OSMR | 0.426928 | 3.14E-24 | 2.23E-21 | 515 |
| B3GNT3 | 0.426539 | 3.49E-24 | 2.33E-21 | 515 |
| RHOF | 0.423512 | 7.87E-24 | 5.07E-21 | 515 |
| ERRFI1 | 0.423174 | 8.61E-24 | 5.38E-21 | 515 |
| PKP3 | 0.421416 | 1.37E-23 | 8.08E-21 | 515 |
| GNA15 | 0.418699 | 2.82E-23 | 1.56E-20 | 515 |
| S100A16 | 0.414672 | 8.05E-23 | 4.35E-20 | 515 |
| FOSL2 | 0.411966 | 1.62E-22 | 8.30E-20 | 515 |
| FAM40B | 0.406823 | 6.00E-22 | 2.93E-19 | 515 |
| ANGPTL4 | 0.406644 | 6.28E-22 | 2.99E-19 | 515 |
| C15orf48 | 0.404011 | 1.22E-21 | 5.41E-19 | 515 |
| IER5 | 0.403517 | 1.38E-21 | 5.98E-19 | 515 |
| PFKP | 0.402417 | 1.81E-21 | 7.39E-19 | 515 |
| CCND1 | 0.402124 | 1.95E-21 | 7.79E-19 | 515 |
| TNFRSF1A | 0.39974 | 3.51E-21 | 1.32E-18 | 515 |
| SBNO2 | 0.399164 | 4.05E-21 | 1.45E-18 | 515 |
| BCL9L | 0.39916 | 4.05E-21 | 1.45E-18 | 515 |
| LTBR | 0.396589 | 7.61E-21 | 2.58E-18 | 515 |
| HDGF | 0.396144 | 8.48E-21 | 2.82E-18 | 515 |
| SULT2B1 | 0.39588 | 9.04E-21 | 2.94E-18 | 515 |
| GJB3 | 0.395336 | 1.03E-20 | 3.22E-18 | 515 |
| RIPK2 | 0.394486 | 1.27E-20 | 3.84E-18 | 515 |
| LOXL2 | 0.393741 | 1.52E-20 | 4.53E-18 | 515 |
| SURF4 | 0.393419 | 1.64E-20 | 4.69E-18 | 515 |
| LAMC2 | 0.393275 | 1.70E-20 | 4.78E-18 | 515 |
| FAM83H | 0.393169 | 1.74E-20 | 4.84E-18 | 515 |
| GOLM1 | 0.39272 | 1.94E-20 | 5.25E-18 | 515 |
| C19orf21 | 0.392549 | 2.02E-20 | 5.34E-18 | 515 |
| KRT6A | 0.392485 | 2.06E-20 | 5.34E-18 | 515 |
| NAMPT | 0.392271 | 2.16E-20 | 5.52E-18 | 515 |
| FOSL1 | 0.39224 | 2.18E-20 | 5.52E-18 | 515 |
| TNFRSF21 | 0.391473 | 2.62E-20 | 6.55E-18 | 515 |
| GALNT2 | 0.389381 | 4.32E-20 | 1.03E-17 | 515 |
| CEBPB | 0.389245 | 4.46E-20 | 1.05E-17 | 515 |
| LAMB3 | 0.384969 | 1.22E-19 | 2.62E-17 | 515 |
| BCL3 | 0.384946 | 1.23E-19 | 2.62E-17 | 515 |
| KRT18 | 0.382329 | 2.27E-19 | 4.43E-17 | 515 |
| C11orf24 | 0.382295 | 2.29E-19 | 4.43E-17 | 515 |
| PLEC | 0.381786 | 2.57E-19 | 4.90E-17 | 515 |
| YWHAZ | 0.381255 | 2.91E-19 | 5.48E-17 | 515 |
| CEACAM19 | 0.381078 | 3.03E-19 | 5.66E-17 | 515 |
| MUC4 | 0.380749 | 3.27E-19 | 6.05E-17 | 515 |
| CDA | 0.378922 | 4.98E-19 | 8.97E-17 | 515 |
| ERO1L | 0.377635 | 6.69E-19 | 1.19E-16 | 515 |
| TNS4 | 0.376672 | 8.33E-19 | 1.47E-16 | 515 |
| GJB2 | 0.376342 | 8.98E-19 | 1.56E-16 | 515 |
| HN1 | 0.37614 | 9.40E-19 | 1.62E-16 | 515 |
| PPP1R14B | 0.375152 | 1.18E-18 | 1.98E-16 | 515 |
| YWHAG | 0.374694 | 1.30E-18 | 2.13E-16 | 515 |
| PLAUR | 0.374666 | 1.31E-18 | 2.13E-16 | 515 |
| PITX1 | 0.371891 | 2.45E-18 | 3.74E-16 | 515 |
| NOTCH3 | 0.371402 | 2.74E-18 | 4.11E-16 | 515 |
| FBXO32 | 0.369333 | 4.34E-18 | 6.38E-16 | 515 |
| KRT6C | 0.36914 | 4.53E-18 | 6.61E-16 | 515 |
| MOCOS | 0.368945 | 4.73E-18 | 6.85E-16 | 515 |
| OPN3 | 0.368751 | 4.94E-18 | 7.05E-16 | 515 |
| PHLDA2 | 0.366667 | 7.82E-18 | 1.10E-15 | 515 |
| TMSB10 | 0.364632 | 1.22E-17 | 1.65E-15 | 515 |
| MYO1E | 0.363471 | 1.57E-17 | 2.07E-15 | 515 |
| RAET1E | 0.363387 | 1.60E-17 | 2.09E-15 | 515 |
| SRD5A3 | 0.358595 | 4.50E-17 | 5.39E-15 | 515 |
| MUC16 | 0.357808 | 5.33E-17 | 6.26E-15 | 515 |
| SH2D3A | 0.356606 | 6.88E-17 | 8.00E-15 | 515 |
| SLC39A1 | 0.356377 | 7.22E-17 | 8.35E-15 | 515 |
| KRT16 | 0.355988 | 7.84E-17 | 9.01E-15 | 515 |
| CHST15 | 0.355674 | 8.38E-17 | 9.52E-15 | 515 |
| SPHK1 | 0.353498 | 1.33E-16 | 1.43E-14 | 515 |
| HHIPL2 | 0.353116 | 1.44E-16 | 1.54E-14 | 515 |
| C19orf28 | 0.353049 | 1.46E-16 | 1.55E-14 | 515 |
| FADD | 0.352402 | 1.67E-16 | 1.76E-14 | 515 |
| GPR97 | 0.352096 | 1.78E-16 | 1.84E-14 | 515 |
| STEAP1 | 0.351804 | 1.89E-16 | 1.95E-14 | 515 |
| PMEPA1 | 0.351616 | 1.97E-16 | 2.02E-14 | 515 |
| SLC2A1 | 0.350267 | 2.60E-16 | 2.63E-14 | 515 |
| ENTPD7 | 0.350004 | 2.75E-16 | 2.75E-14 | 515 |
| FLJ36031 | 0.349943 | 2.78E-16 | 2.77E-14 | 515 |
| P2RY6 | 0.34928 | 3.19E-16 | 3.11E-14 | 515 |
| CEP55 | 0.349216 | 3.24E-16 | 3.12E-14 | 515 |
| C8orf76 | 0.348915 | 3.45E-16 | 3.28E-14 | 515 |
| SLC7A5 | 0.348459 | 3.79E-16 | 3.57E-14 | 515 |
| C17orf53 | 0.348282 | 3.93E-16 | 3.63E-14 | 515 |
| PAEP | 0.348261 | 3.94E-16 | 3.63E-14 | 515 |
| ACTG1 | 0.346906 | 5.21E-16 | 4.69E-14 | 515 |
| PTK6 | 0.346652 | 5.49E-16 | 4.92E-14 | 515 |
| ANLN | 0.346585 | 5.56E-16 | 4.94E-14 | 515 |
| LY6D | 0.346385 | 5.80E-16 | 5.10E-14 | 515 |
| GALNT4 | 0.34625 | 5.96E-16 | 5.22E-14 | 515 |
| LDHA | 0.345888 | 6.42E-16 | 5.53E-14 | 515 |
| SHC1 | 0.343926 | 9.57E-16 | 7.81E-14 | 515 |
| SLC24A6 | 0.342746 | 1.22E-15 | 9.68E-14 | 515 |
| S100A11 | 0.34269 | 1.23E-15 | 9.71E-14 | 515 |
| GNPNAT1 | 0.342187 | 1.36E-15 | 1.07E-13 | 515 |
| ITPKA | 0.338312 | 2.96E-15 | 2.18E-13 | 515 |
| PITPNC1 | 0.337951 | 3.18E-15 | 2.32E-13 | 515 |
| MDFI | 0.337847 | 3.24E-15 | 2.35E-13 | 515 |
| GPR172A | 0.337522 | 3.46E-15 | 2.49E-13 | 515 |
| TK1 | 0.33672 | 4.05E-15 | 2.87E-13 | 515 |
| TPM3 | 0.336703 | 4.07E-15 | 2.87E-13 | 515 |
| CARD14 | 0.336643 | 4.11E-15 | 2.90E-13 | 515 |
| UHRF1 | 0.336605 | 4.15E-15 | 2.91E-13 | 515 |
| HMGA1 | 0.336224 | 4.47E-15 | 3.11E-13 | 515 |
| RAB10 | 0.336188 | 4.50E-15 | 3.12E-13 | 515 |
| F3 | 0.335184 | 5.49E-15 | 3.77E-13 | 515 |
| LOC100132111 | 0.335011 | 5.68E-15 | 3.87E-13 | 515 |
| ARPC1B | 0.334757 | 5.97E-15 | 4.03E-13 | 515 |
| ITGA3 | 0.334396 | 6.40E-15 | 4.28E-13 | 515 |
| RAB27B | 0.334339 | 6.47E-15 | 4.31E-13 | 515 |
| AVEN | 0.334212 | 6.64E-15 | 4.37E-13 | 515 |
| OAS1 | 0.33405 | 6.85E-15 | 4.48E-13 | 515 |
| C19orf33 | 0.333949 | 6.99E-15 | 4.54E-13 | 515 |
| IER5L | 0.333941 | 7.00E-15 | 4.54E-13 | 515 |
| CD109 | 0.333898 | 7.06E-15 | 4.57E-13 | 515 |
| FUT3 | 0.333319 | 7.90E-15 | 5.06E-13 | 515 |
| C11orf86 | 0.332616 | 9.07E-15 | 5.75E-13 | 515 |
| FAM129B | 0.332399 | 9.46E-15 | 5.93E-13 | 515 |
| MYH16 | 0.331966 | 1.03E-14 | 6.41E-13 | 515 |
| ITGB4 | 0.331365 | 1.16E-14 | 7.07E-13 | 515 |
| ARHGEF4 | 0.331031 | 1.23E-14 | 7.45E-13 | 515 |
| EPHB4 | 0.329768 | 1.57E-14 | 9.28E-13 | 515 |
| GPRC5A | 0.329667 | 1.61E-14 | 9.44E-13 | 515 |
| PVRL2 | 0.329215 | 1.75E-14 | 1.02E-12 | 515 |
| C16orf57 | 0.328472 | 2.02E-14 | 1.17E-12 | 515 |
| KRT6B | 0.328392 | 2.05E-14 | 1.18E-12 | 515 |
| DPP9 | 0.328047 | 2.19E-14 | 1.25E-12 | 515 |
| HAL | 0.327949 | 2.23E-14 | 1.28E-12 | 515 |
| C9orf140 | 0.327815 | 2.29E-14 | 1.30E-12 | 515 |
| HEATR2 | 0.327484 | 2.44E-14 | 1.37E-12 | 515 |
| SPDEF | 0.327294 | 2.53E-14 | 1.42E-12 | 515 |
| DUSP4 | 0.327051 | 2.65E-14 | 1.48E-12 | 515 |
| CDH3 | 0.326949 | 2.70E-14 | 1.50E-12 | 515 |
| PPAPDC1A | 0.326518 | 2.93E-14 | 1.62E-12 | 515 |
| SLC6A14 | 0.326502 | 2.94E-14 | 1.62E-12 | 515 |
| ITGB1 | 0.326419 | 2.99E-14 | 1.64E-12 | 515 |
| PACS1 | 0.326213 | 3.11E-14 | 1.70E-12 | 515 |
| PPM1G | 0.325918 | 3.29E-14 | 1.79E-12 | 515 |
| RHBDL2 | 0.325778 | 3.38E-14 | 1.83E-12 | 515 |
| TRIP10 | 0.325311 | 3.69E-14 | 1.98E-12 | 515 |
| SERPINB1 | 0.3249 | 3.99E-14 | 2.13E-12 | 515 |
| MLKL | 0.324319 | 4.45E-14 | 2.35E-12 | 515 |
| MRPL19 | 0.322746 | 5.99E-14 | 3.11E-12 | 515 |
| SC65 | 0.322573 | 6.19E-14 | 3.20E-12 | 515 |
| RGS20 | 0.322221 | 6.61E-14 | 3.36E-12 | 515 |
| GAPDH | 0.321358 | 7.77E-14 | 3.90E-12 | 515 |
| TUBA1C | 0.321336 | 7.80E-14 | 3.91E-12 | 515 |
| S100P | 0.321292 | 7.86E-14 | 3.93E-12 | 515 |
| SLC27A4 | 0.320822 | 8.58E-14 | 4.26E-12 | 515 |
| LRFN4 | 0.320334 | 9.40E-14 | 4.62E-12 | 515 |
| COL1A1 | 0.320028 | 9.95E-14 | 4.86E-12 | 515 |
| GREM1 | 0.319102 | 1.18E-13 | 5.72E-12 | 515 |
| PYCR1 | 0.319042 | 1.20E-13 | 5.76E-12 | 515 |
| KCMF1 | 0.31885 | 1.24E-13 | 5.94E-12 | 515 |
| DSG2 | 0.318644 | 1.29E-13 | 6.12E-12 | 515 |
| IL31RA | 0.318577 | 1.30E-13 | 6.18E-12 | 515 |
| CFL1 | 0.317874 | 1.48E-13 | 6.90E-12 | 515 |
| NRBP1 | 0.3178 | 1.50E-13 | 6.94E-12 | 515 |
| MRPL15 | 0.31755 | 1.57E-13 | 7.25E-12 | 515 |
| TRIM47 | 0.317404 | 1.62E-13 | 7.41E-12 | 515 |
| TMEM214 | 0.316944 | 1.76E-13 | 8.00E-12 | 515 |
| VDAC2 | 0.316937 | 1.76E-13 | 8.00E-12 | 515 |
| LOC100270710 | 0.316928 | 1.77E-13 | 8.00E-12 | 515 |
| FURIN | 0.316656 | 1.86E-13 | 8.39E-12 | 515 |
| GORASP2 | 0.316531 | 1.90E-13 | 8.55E-12 | 515 |
| LPGAT1 | 0.316155 | 2.03E-13 | 9.00E-12 | 515 |
| TNFRSF10B | 0.315189 | 2.43E-13 | 1.06E-11 | 515 |
| SPINT1 | 0.31382 | 3.11E-13 | 1.34E-11 | 515 |
| DERL1 | 0.313702 | 3.18E-13 | 1.36E-11 | 515 |
| CTBP2 | 0.313668 | 3.20E-13 | 1.37E-11 | 515 |
| EIF2C2 | 0.313378 | 3.37E-13 | 1.43E-11 | 515 |
| PABPC1 | 0.313212 | 3.48E-13 | 1.47E-11 | 515 |
| OAS3 | 0.312949 | 3.65E-13 | 1.54E-11 | 515 |
| COL11A1 | 0.312769 | 3.77E-13 | 1.58E-11 | 515 |
| S100A10 | 0.31234 | 4.07E-13 | 1.70E-11 | 515 |
| IER3 | 0.311901 | 4.41E-13 | 1.82E-11 | 515 |
| TUBA4A | 0.311688 | 4.58E-13 | 1.88E-11 | 515 |
| KRT19 | 0.311561 | 4.69E-13 | 1.92E-11 | 515 |
| SSH3 | 0.311448 | 4.78E-13 | 1.95E-11 | 515 |
| STK24 | 0.310531 | 5.64E-13 | 2.28E-11 | 515 |
| CLIP1 | 0.310097 | 6.10E-13 | 2.46E-11 | 515 |
| JUP | 0.309928 | 6.28E-13 | 2.51E-11 | 515 |

Supplementary Table 2. The transcript sequence for AC068228.1

ENST00000522383.1 (AC068228.1) length=777

GAACACACCACGGAGGAGTGGGGCTCCCGCACCCCCGGGAGGCCGCCTGCTCCCAGGACCGGCGTGCAGGGCACCTCCAAGAAGTGATTTACCGCTGTCCTGAGCAATGCTTCTCAGAACTCCTCATCTGCAGCTGCGGGATTGGCTTATCTCTCAGGGCCACCTGCCCTGCCCTCGGGCCGGGAGGGCTGGCTCAGACACCTCCTTGGCACAGTCAGCCATGGGATATTTCCTCTCCTCCCAGCCGCAGGGGGAGGCGAAGAGACCGCAGAGGTTCCAGTTCTCAGGCCAACGCTTCCTCGCTGGAAGTGGCTTCTTGGAACACTGTCTGGAGAACGAGCCGAGGCTGCCGGCGGCCCACGAGCCCAGGGGACCAGTCAGTGTTGGCGCTGGAGTGAGAGGAGCAGCACGTGGGTTTGAAATCTATGAACTCTGAGGGGAGTGACCACGTGTTCTTTCCAGATCAGATCGTGATCTGGCAATGGGATGGACTGCCTAGAACCCTGACTCTCTTTGGGTGGGCTCTGCTGGAAACAGGCTCTGAGACTAGGATTTCAGTACAAGTGGTTGGTCGGAAGATCATTCTGAGAAGTACAGAGAGAAGTGGGGAAGAGAAACGGGAAGGGAAGGAAGCCAGGCCAGGTGAAGTTGAGGAGCAGGCCACTGCTGTGGACATCTGGGTTCATGGACATCTCTGGGGACATCAGGGGGTTCCAGCCTGTGGGAACCTCTGGGTGATGGTGCCAAATATATCTCAAGGTTTTCCTGCCCAAAGGG
